# Recurrent and Prolonged Infections in a Child with a Homozygous *IFIH1* Nonsense Mutation

**DOI:** 10.3389/fgene.2017.00130

**Published:** 2017-09-22

**Authors:** Maha Zaki, Michaela Thoenes, Amit Kawalia, Peter Nürnberg, Rolf Kaiser, Raoul Heller, Hanno J. Bolz

**Affiliations:** ^1^Human Genetics and Genome Research Division, Clinical Genetics Department, National Research Centre Cairo, Egypt; ^2^Institute of Human Genetics, University Hospital of Cologne Cologne, Germany; ^3^Cologne Center for Genomics, University of Cologne Cologne, Germany; ^4^Cologne Excellence Cluster on Cellular Stress Responses in Aging-Associated Diseases, University of Cologne Cologne, Germany; ^5^Institute of Virology, University Hospital of Cologne Cologne, Germany

**Keywords:** *IFIH1*, immunodeficiency, consanguinity, infection, loss-of-function, whole-exome sequencing

## Abstract

In an Egyptian girl born to consanguineous parents, whole-exome sequencing (WES) identified a homozygous mutation in *PHGDH*, c.1273G>A (p.Val425Met), indicating 3-phosphoglycerate dehydrogenase deficiency. This diagnosis was compatible with the patient’s microcephaly, severe psychomotor retardation, seizures and cataracts. However, she additionally suffered from recurrent (at least monthly) episodes of prolonged and severe chest infections requiring hospitalization, suggesting a secondary, predisposing and potentially Mendelian, condition. A local reactivation of an EBV infection in the respiratory tract was detected after a recent chest infection, likely representing an opportunistic infection based on a compromised immune system. Further inspection of WES data revealed a homozygous nonsense mutation, c.2665A>T (p.Lys889^∗^), in *IFIH1*, encoding MDA5. MDA5 detects long viral double-stranded RNA that is generated during replication of picorna viruses, and thereby activates the type I interferon signaling pathway. The results of Western blot analysis of protein from cultured fibroblasts of the patient indicates absence of wild type MDA5/IFIH1, compatible with NMD. We propose that, analogous to the severe course of primary influenza infection due to biallelic deficiency of a downstream effector, IRF7, homozygous loss of *IFIH1* defines a novel Mendelian immunodeficiency disorder that increases susceptibility to severe viral infections. This is contrasted to heterozygous gain-of-function *IFIH1* mutations in autoimmune diseases. Our findings highlight the potential of comprehensive genomic investigations in patients from consanguineous families to identify monogenic predispositions to severe infections.

## Introduction

Parental consanguinity enhances the risk for genetic disorders in the offspring due to homozygous mutations in recessive disease genes. Additional symptoms beyond the usual manifestations of the diagnosed entity may reflect phenotypic variability, but can also be due to homozygosity for mutations at additional loci. By whole-exome sequencing (WES) and filtering for homozygous loss-of-function variants, we have disentangled the complex phenotype of an Egyptian girl born to first-cousin parents. Her phenotype results from simultaneous 3-PHGDH deficiency and a novel recessive immunodeficiency syndrome due to a homozygous *IFIH1* nonsense mutation (Supplementary Table S1). Such loss-of-function (LoF) variants represent a (probably underestimated) group of Mendelian predispositions rather than disorders: they do not *per se* cause disease, but, depending on the exposure to external trigger factors, they strongly predispose to non-genetic disease (such as infections). Our findings demonstrate the diagnostic potential of whole-exome and -genome sequencing in severely ill pediatric patients. Careful assessment and alignment of clinical findings with genomic data is crucial and may unlock novel rare entities from “by-catch” data in apparently solved cases with “plus symptoms.”

### Patient

The study was approved by the institutional review boards of the Ethics Committee of the University Hospital of Cologne and the National Research Centre, Cairo. Informed consent for genetic investigations and publication of facial images was obtained from the parents. Clinical and specimen investigations were conducted according to the Declaration of Helsinki.

The parents of the index patient and her healthy siblings (both minors) received detailed genetic counseling, including all information about our study aimed at the identification of the genetic cause of the patient’s severe condition.

Our patient, II:1, is a girl of 5^8^/_12_ years (see Supplementary Figure S1 for clinical presentation). Pregnancy and birth history were uneventful. Microcephaly was noted at birth (head circumference of 30 cm, -2.5 SD). Patient II:1 was first seen at the National Research Centre, Neurogenetics Clinic, at the age of 2 years. At that time, she had not acquired any motor or cognitive milestones. For example, she was not able to lift her head or visually follow objects. Seizures occurred since the age of 2 months and were initially myoclonic. Over time, she developed tonic seizures with cyanosis, sometimes evolving into generalized tonic-clonic seizures. EEG revealed hypsarrhythmia. The epilepsy was treated with valproate and leviteracetam. She had axial and limb hypertonia and brisk reflexes. Bilateral congenital cataract was treated by cataract extraction at 2^3^/_12_ years, but did not lead to noticeable improvements (persistent inability to follow objects). Furthermore, ophthalmological evaluation revealed a Marcus Gunn phenomenon (trigemino-oculomotor synkinesis) induced by suckling. Patient II:1 has continuously been followed up by us since her first examination in our clinic. She still is profoundly delayed and lacks any milestones. Seizures are fairly controlled by clonazepam, occurring once or twice per day with mild myoclonia. Physiotherapy has not been applied over the past 2 years, and severe spasticity and arthrogryposis have developed during that time. The current head circumference is 40 cm (-8.5 SD), weight is 10 kg (-3.3 SD), and length is 90 cm (-5.9 SD). Brain MRI showed simplified gyral pattern, a hypoplastic corpus callosum and normal brain stem and cerebellum (Supplementary Figures S1d,e). From early on, frequent episodes of high fever, mostly related to severe upper and lower respiratory tract/chest infections, have required hospitalization with antiviral and antibacterial medication at least twice a month, sometimes in the Pediatric Intensive Care Unit. Fever episodes were initially associated with seizures and rapid deterioration, raising the suspicion of bacterial encephalitis which could not be confirmed by lumbar puncture. The treatment therefore consisted of both antiviral (including Acyclovir, Amantadine, Ganciclovir, and Isoprinosine) and antibacterial therapy. Results from echocardiography and abdominal ultrasound were normal. No skin changes were noted. Chromosome analysis revealed a normal female karyotype (46,XX). Apart from elevated IgG3 and decreased IgG4 levels, results from blood count and immunological evaluation were normal (Supplementary Table S2).

**FIGURE 1:**
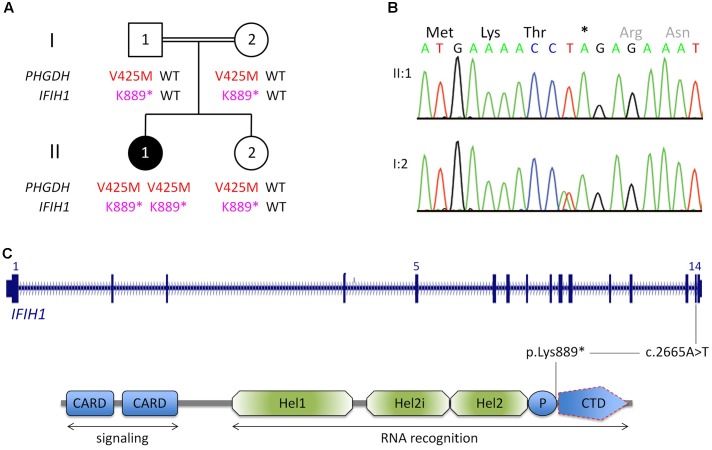
Genetic analysis. **(A)** Pedigree of the Egyptian family reported herein. The index patient, II:1, is affected by 3-phosphoglycerate dehydrogenase deficiency, as indicated by homozygosity for a previously reported LoF mutation in *PGHDH*, p.Val425Met. In addition, she is homozygous for a nonsense mutation in *IFIH1*. (For brevity, the one-letter-code has been applied in the pedigree scheme.) **(B)** Electropherograms of the *IFIH1* mutation [index patient, upper panel; heterozygous carrier (mother), lower panel]. **(C)** Scheme of *IFIH1* gene and protein. The nonsense mutation (c.2665A>T/p.Lys889^∗^) resides in exon 14. If c.2665A>T was compatible with production of IFIH1 protein, it would lack the C-terminal domain (CTD), which is responsible for binding viral dsRNA. However, the lack of IFIH1 protein in patient fibroblasts (**Figure 2**) indicates that the homozygous nonsense mutation prevents IFIH1 protein production. Hel1, Hel2i, P, CTD. CARD, caspase activation recruitment domain; Hel, helicase domain; Hel1 and Hel2, the two conserved core Hel domains; Hel2i, insertion domain that is conserved in the RIG-I-like family; P, pincer/bridge region connecting Hel2 to the CTD.

The parents are healthy first cousins, and the patient’s sister, II:2, is healthy and 1^4^/_12_ years old (**Figure 1A**). There is no family history of conditions corresponding to the symptoms of II:1.

### Virology, Protein Studies

All virus tests were negative in nasal swabs and in EDTA blood, except EBV which was positive in nasal swab, indicative for an EBV infection. A false positive result by blood contamination of the swab is excluded because the corresponding blood analysis was negative for EBV. Subsequent EBV antibody profiling was VCA IgG-positive, VCA IgM-negative, and EBNA 1 positive.

**FIGURE 2:**
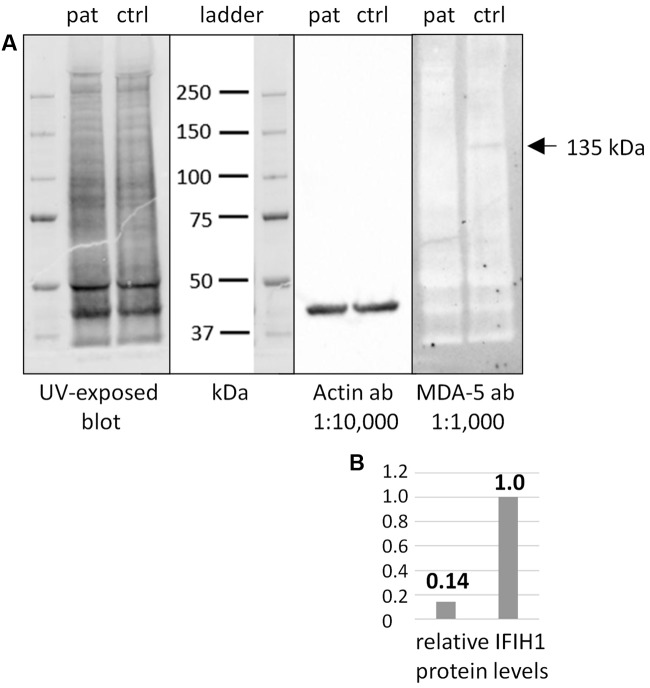
Western blot of IFIH1 using the rabbit monoclonal antibody MDA-5 (D74E4). **(A)** The protein extract from cultured fibroblasts of a healthy control proband (ctrl) yielded a specific band of endogenous IFIH1 at the expected size of 135 kDa. **(B)** The 135 kDa band was reduced to background level (ratio of 1:0.14) in the protein sample from cultured fibroblasts of the patient (pat) indicating the absence of wild type IFIH1. The blot was rehybridized with an anti-actin antibody to adjust signal volumes for equal protein loading between control and patient. According to the manufacturer, MDA-5 mAb recognizes the residues surrounding p.Arg470 of human MDA5 (IFIH1) protein.

Additional functional studies on immune responsiveness based on patient’s cells could not be organized due to logistic reasons. However, we could obtain a skin biopsy from the patient and derived fibroblast cell lines from it. Western blot analysis of MDA5 protein from these cells indicated nonsense-mediated decay (NMD; **Figure 2**).

### Whole-Exome Sequencing

Whole-exome sequencing (mean coverage of 78-fold (30-fold for 82%, and 10-fold for 97% of target sequences) identified 49 rare homozygous candidate variants in 49 genes (Supplementary Table S1). Most variants had documented allele frequencies in the general population (including 13 that were found in homozygous state and one (X-linked) in hemizygous state in at least one healthy individual, ExAC database) or low pathogenicity values in pathogenicity prediction programs, or both. Variants in genes associated with unrelated human diseases (*HSPG2*, *LOR*, *IGSF10*, *CACNA1H*, *SLC46A1*, *TBX4*, *RGS9*, *TMC6*, *ACTN4*, *KIF4A*, *XIAP*) and unrelated or unremarkable phenotypes of knockout mice (*NOL9*, *RERE*, *SPEN*, *ARHGEF11*, *IGSF9*, *CD244*, *KIF21B*, *PLA2R1*, *SEMA5B*, *ACSL1*, *FOXQ1*, *CSPG4*, *ABCC1*, *KIFC3*, *HELZ*, *GALR3*) largely excluded several genes as potential candidate disease genes. One variant, c.1273G>A (p.Val425Met) in *PHGDH*, is a known recessive disease-causing mutation (Klomp et al., 2000; Tabatabaie et al., 2009) and explains most of the symptoms of patient II:1. Bioinformatic software predicted pathogenicity of p.Val425Met*_PHGDH_*, and segregation analysis was compatible with a causative role of the mutation (**Figure 1A**). The p.Val425Met*_PHGDH_* mutation has been documented in the general population (rs121907988), but at very low frequency (MAF of 0.001%; ExAC database) and not in homozygous state. Searching the WES data of patient II:1 for putative loss-of-function (LoF) variants in immunity-related genes revealed a homozygous nonsense variant, c.2665A>T (p.Lys889^∗^), in *IFIH1*, which was absent from the ExAC and the 1TGP databases. As in the case of the *PHGDH* mutation, p.Ly889^∗^*_IFIH1_* was present in heterozygous state in the healthy sister of patient II:1, and in both parents (**Figures 1A,B**).

## Background

*IFIH1* encodes MDA5, a cytosolic double-stranded viral RNA (dsRNA) sensor. Upon binding of viral long dsRNA, MDA5 triggers the signaling molecules IPS-1, IRF3 and IRF7, causing transcription of the antiviral type 1 interferon (IFN). This induces transcription of 100s of IFN-stimulated genes to contain the infection. Several viruses have developed gene functions that decrease IFIH1/MDA5 activity, indicating its central role in antiviral responses (Ivashkiv and Donlin, 2014; del Toro Duany et al., 2015).

Heterozygous *gain*-of-function mutations of *IFIH1* cause autosomal dominant autoimmune disorders, namely systemic lupus erythematosus (SLE) in mice (Funabiki et al., 2014), and SLE, Aicardi-Goutieres syndrome (AGS) or Singleton-Merten syndrome (SMS) in humans (Oda et al., 2014; Rice et al., 2014; Rutsch et al., 2015; Van Eyck et al., 2015). No recessive phenotype had been linked to *IFIH1* mutations at the time of submission (see Concluding Remarks).

## Discussion

The *PHGDH* mutation in patient II:1, c.1273G>A (p.Val425Met), has been reported previously and results in almost undetectable activity of the encoded enzyme, 3-phosphoglycerate dehydrogenase (3-PHGDH; MIM# 606879) (Klomp et al., 2000; Tabatabaie et al., 2009). PHGDH catalyzes the first committed step in the biosynthesis of L-serine, an important metabolite involved in various processes such as biosynthesis of proteins, phospholipids, sphingomyelin, cysteine, purines, thymidine, and neuromodulators (D-serine and glycine). Because L-serine (which can be derived from dietary intake and degradation of proteins and phospholipids) does not easily cross the blood–brain barrier (Smith et al., 1987), its endogenous *de novo*-synthesis is essential for brain development and function. 3-PHGDH deficiency (MIM# 601815) is a rare autosomal recessive disorder characterized by congenital microcephaly, psychomotor retardation and intractable seizures. Impaired brain development is reflected by delayed myelinization and white matter abnormalities, and some patients have cataracts (Jaeken et al., 1996; Klomp et al., 2000). Although endogenous L-serine synthesis is essential, there has been evidence that neurological symptoms may be alleviated by supplementation commenced during pregnancy (orally given to the mother) (de Koning, 2006). Homozygosity for p.Val425Met*_PHGDH_* is compatible with most of the phenotype of patient II:1 (Supplementary Figure S1). However, although chronically and severely ill children are more prone to infections than healthy contemporaries, patient II:1 has a striking history of recurrent infections, which is not typical for 3-PHGDH deficiency. Searching the WES data for additional homozygous variants identified a rare homozygous nonsense mutation, p.Lys889^∗^, in *IFIH1*, a likely cause for the enhanced susceptibility to infections.

Autosomal dominant inheritance has been postulated (Falls et al., 1949) for the Marcus Gunn phenomenon (trigemino-oculomotor synkinesis; MIM #154600), but no gene has convincingly been associated with this manifestation. In patient II:1, it is probably unrelated to the recessive mutations in *PHGDH* and *IFIH1* and may represent a third genetic condition, perhaps due to a *de novo* mutation.

The nonsense *IFIH1* nonsense mutation of patient II:1, p.Lys889^∗^, resides in the second to last exon, exon 14, and is predicted to result either in unstable RNA subjected to NMD or in a truncated protein lacking the C-terminal domain, CTD (**Figure 1C**). The CTD binds the ds viral RNA and is therefore essential for MDA5 function (del Toro Duany et al., 2015). Both protein truncation or NMD due to p.Lys889^∗^ allele can be regarded as loss-of-function. The results of Western blot analysis of protein from cultured fibroblasts of the patient indicate absence of wild type IFIH1 (**Figure 2**), compatible with NMD.

So far, no human disorder has been linked to biallelic recessive *IFIH1* mutations, but *Ifih1*^-/-^ knockout mice show increased mortality when infected with West Nile virus (WNV), indicating impaired immune defense (Errett et al., 2013). Strikingly, compound heterozygous null mutations of *IRF7*, encoding interferon regulatory factor 7 which is triggered by MDA5, have been shown to underlie severe influenza in an otherwise healthy child (Ciancanelli et al., 2015). In view of this highly analogous constellation, it is unlikely that sole homozygosity for the *IFIH1* nonsense mutation would have led to congenital symptoms (unrelated to infections) in patient II:1. However, because she is simultaneously affected by congenitally manifesting PHGDH deficiency, we cannot determine this with certainty.

The heterozygous *IFIH1* missense mutations underlying several autosomal dominant autoimmune disorders, namely SLE, AGS and SMS, enhance interferon beta induction by MDA5, opposed to the *IFIH1* nonsense mutation of patient II:1 which can be considered recessive (heterozygous carriers – the parents and the patient’s sister – are healthy). Genome-wide association (GWA) studies have shown that supposedly activating *IFIH1* variants – analogous to fully penetrant gain-of-function mutations underlying the dominant Mendelian diseases AGS and SMS – are implicated in the pathogenesis of autoimmune disorders such as type 1 diabetes (Nejentsev et al., 2009) and SLE (Molineros et al., 2013). In contrast, certain supposed LoF SNPs in *IFIH1*, including the nonsense variant p.Glu627^∗^ (rs35744605), confer protection against type 1 diabetes. Consistent with its protective role, the ExAC database annotates a relatively high MAF for rs35744605 (0.33%) – in contrast to the extreme rarity of AGS- and SMS-causing mutations (only 1/11 HGMD-listed mutations is ExAC-annotated at all), but also in contrast to the un-annotated LoF mutation p.Lys889^∗^ reported herein. Homozygosity of rs35744605 is listed for only 1/60,000 healthy individuals, indicating that biallelic LoF may be disadvantageous (and hence rare in healthy cohorts) due to increased vulnerability for viral infections. On the other hand, biallelic *IFIH1* LoF mutations may be enriched in the entirety of infants with complicated courses of viral infections. They should be considered in such cases, and this appears even more advisable in densely populated areas (where contagious viral infections are easily transmitted) of countries with high rates of consanguinity (where risk for homozygosity for very rare LoF alleles like p.Lys889^∗^ is enhanced), such as Cairo where patient II:1 is from.

Samples for PCR-based virus detection were available from patient II:1 only after an episode of a milder chest infection that did not require hospitalization. No picorna viruses were detectable, but an active infection with a DNA virus, EBV, was identified. The antibody profile indicated a reactivated EBV infection. Primary EBV infections can occur in childhood, adolescence (as infectious mononucleosis) or reactivate from latency (EBV is, like all herpesvirus infections which are usually controlled by the immune system, a persisting virus infection). If the immune system is compromised, both primary and reactivated EBV infection may be more severe and prolonged. This predisposition to EBV disease has been shown for several primary immunodeficiencies caused by mutations in various genes (Cohen, 2015; Svobodova et al., 2015). EBV-based pulmonary/respiratory infections have been reported (Miron et al., 2002; Svobodova et al., 2015), but they are rare, especially in reactivated infections. In patient II:1, the reactivation of the EBV infection occurred locally, in the respiratory system (EBV was found in the nasal swab, but not in the blood sample). It is very likely that the EBV reactivation is responsible for the recent chest infection of patient II:1, representing an opportunistic infection based on her compromised immune system with frequent infections with picorna viruses, e.g., RSV (due to her *IFIH1* deficiency with impaired sensing of infections with dsRNA, but not DNA viruses). Repeated testing for picorna viruses in patient II:1 during different infection periods was not possible so far, and detailed parameters of the patient’s immunity were not available. This would be needed to determine the causative agent in future episodes of acute and severe infection and to further substantiate our hypothesis of monogenic immunodeficiency due to *IFIH1* deficiency.

## Concluding Remarks

*IFIH1* deficiency adds to an as yet short list of autosomal recessively inherited single-gene defects that predispose to infectious disease. Another recent example are biallelic mutations of *DOCK2*, encoding human dedicator of cytokinesis 2, leading to combined immunodeficiencies and early-onset invasive infections (Dobbs et al., 2015). While our manuscript was under review, two independent studies reported increased susceptibility to respiratory viral infections due to biallelic *IFIH1* mutations (Asgari et al., 2017; Lamborn et al., 2017), supporting our findings. With novel sequencing technologies having entered this field of research, several other monogenic defects causing enhanced vulnerability to infections are likely to be uncovered in the near future.

Diagnostic rapid whole exome- and genome sequencing are increasingly being applied in newborns with certain clinical findings, such as congenital malformations, syndromic conditions, and assumingly inherited disorders in order to avoid extensive diagnostic processes (Saunders et al., 2012; Smith et al., 2016). We propose to add severe and prolonged pediatric infections as an indication for such approaches. Mendelian predispositions to infectious diseases may be underestimated, and early molecular diagnosis is important for individual management and prophylaxis.

## Author Contributions

MZ, RH, PN, AK, RK, and HJB substantially contributed to the conception and design of the work. MT carried out the lab experiments. AK and PN performed bioinformatic analyses of NGS data. All authors analyzed and interpreted the data for the work. All authors drafted the work, revised it critically for important intellectual content and finally approved the version to be published. They all agreed to be accountable for all aspects of the work in ensuring that questions related to the accuracy or integrity of any part of the work are appropriately investigated and resolved.

## Conflict of Interest Statement

The authors declare that the research was conducted in the absence of any commercial or financial relationships that could be construed as a potential conflict of interest.
