# Supplementary material for: Recurrent and Prolonged Infections in a Child with a Homozygous IFIH1 Nonsense Mutation
Source: Front Genet. 2017 Sep 22;8:130. doi: 10.3389/fgene.2017.00130 (PMC5614965; doi:10.3389/fgene.2017.00130)
Supplement: Supplementary file 1 [file Data_Sheet_1.docx]

Supplementary Material

Recurrent and prolonged infections in a child with a homozygous *IFIH1* nonsense mutation

**Maha Zaki^1^, Michaela Thoenes^2^, Amit Kawalia^3^, Peter Nürnberg^3,4^, Rolf Kaiser^5^, Raoul Heller^2^, Hanno J. Bolz^2^***

*** Correspondence:** Hanno J. Bolz: hanno.bolz@uk-koeln.de

# Supplementary Data

**1.1 Material and Methods**

All methods were carried out in accordance with the approved guidelines.

**Patients**

The study was approved by the institutional review boards of the Ethics Committee of the University Hospital of Cologne and the National Research Centre, Cairo. Informed consent for genetic investigations and publication of facial images was obtained from the parents. Clinical and specimen investigations were conducted according to the Declaration of Helsinki.

**Virology**

The respiratory agents were investigated from nasal swab and whole EDTA blood which had been obtained 10 days after the patient had been seen at the outpatients clinic with symptoms of another chest infection. We used a commercial multiplex PCR-based assay (NxTAG RPP, Luminex B.V. Oosterhout, The Netherlands). This assay amplifies a number of viral and bacterial targets with a mixture of target-specific primers and probes. After amplification, the PCR products are hybridized to different beads, each emitting a different fluorescent light according to the viral or bacterial target listed below. The amplicons are hybridized to the different beads via the specific probe, so that the emitting light is directly correlated to the target. Targets include all relevant picorna viruses and are as follows: Influenza A + B (H3N2, H1N1, H1N1nv), RSV (A+B), hMPV, Parainfluenza 1 – 4, Adenovirus, Coronavirus (229E, OC43, NL63, HKU1), Rhinovirus and Enterovirus (including ECHO, Coxsackie, Entero 68, 71), Bocavirus, Mycoplasma pneumoniae, Chlamydophila pneumoniae, Legionella pneumophila. In addition, testing for CMV, EBV, HSV and VZV was performed with Altona Diagnostics Real Star (Altona Diagnostics, Hamburg, Germany). The eluate was used from a single preparation for respiratory agents and the above mentioned Herpesviruses. The amplicons were analysed with the ROCHE Lightcycler 480. Upon EBV detection from nasal swab, EBV antibody profiling was carried out, comprising virus capsid antigen (VCA) IgG, VCA IgM, and EBV-specific nuclear antigen 1 (EBNA 1).

**Whole-exome sequencing**

Genomic DNA of II:4 was subjected to whole-exome sequencing, WES. Exome capture was performed using the Agilent SureSelectXT Human All Exon 50 Mb kit following manufacturer’s procedures (Agilent, Santa Clara, CA, USA) and sequenced with Illumina paired end sequencing (protocol v1.2). Briefly, DNA was sheared by fragmentation (Covaris, Woburn, MA, USA) and purified using Agencourt AMPure XP beads (Beckman Coulter, Fullerton, CA, USA). Resulting fragments were analysed using an Agilent 2100 Bioanalyzer. Fragment ends were repaired and adaptors were ligated to the fragments. The library was purified using Agencourt AMPure XP beads and amplified by PCR before hybridisation with biotinylated RNA baits. Bound genomic DNA was purified with streptavidin coated magnetic Dynabeads (Invitrogen, Carlsbad, CA, USA) and re-amplified to include barcoding tags before pooling for sequencing on an paired-end, 100 cycle run on an Illumina HiSeq 2000 according to manufacturer’s protocols. Briefly, primary data were filtered according to signal purity by the Illumina Realtime Analysis (RTA) software v1.8. Subsequently, reads were mapped to the human genome reference build hg19 using the bwa-aln ([Li and Durbin, 2009](#_ENREF_7)) alignment algorithm. GATK v1.6 ([McKenna et al., 2010](#_ENREF_9)) was used to mark duplicated reads, for local realignment around short insertions and deletions, to recalibrate the base quality scores and to call SNPs (incorporating variant quality score recalibration) and short indels ([Kawalia et al., 2015](#_ENREF_5)). Scripts developed in-house at the Cologne Center for Genomics were used to detect protein changes, affected donor and acceptor splice sites, and overlaps with known variants. Acceptor and donor splice site mutations were analyzed with a Maximum Entropy model ([Yeo and Burge, 2004](#_ENREF_12)) and filtered for effect changes. In particular, and because the patients came from a consanguineous background, we filtered for high-quality (coverage >15; quality >25) rare (MAF<0.005) homozygous variants (dbSNP build 135, the database of the 1000 Genomes Project build 20110521, TGP ([Via et al., 2010](#_ENREF_11))), and the Exome Variant Server, NHLBI Exome Sequencing Project, Seattle, build ESP6500 ([Fu et al., 2013](#_ENREF_4))). We also filtered against an in-house-database containing all variants from 511 exomes from epilepsy patients to exclude pipeline-related artifacts/false positives (MAF<0.004). The resulting list of candidate genes was then prioritized considering predictions from various bioinformatics tools, (putative) functions of the gene products, and phenotypic data from animals and humans associated with variants in the respective gene, if available (Supplementary Table S1). In addition to the above large-scale sequencing databases consulted, a local pipeline ([Kawalia et al., 2015](#_ENREF_5)) and interface was used (Varbank v.2.3; <https://varbank.ccg.uni-koeln.de>) as described previously ([Beck et al., 2014](#_ENREF_1);[Elsayed et al., 2015](#_ENREF_2)), and we searched the Exome Aggregation Consortium (ExAC) database (Cambridge, MA; <http://exac.broadinstitute.org>, as of 05/2016), which aggregates numerous databases including the current versions of the ESP and the TGP, for the homozygous candidate variants from the mapped regions. Segregation analysis for the mutations identified in *PHGDH* (NM_006623.3; MIM *606879) and *IFIH1* (NM_022168.2; MIM *606951) was carried out by Sanger sequencing.

**Western blot analysis**

After skin biopsy, primary fibroblasts from the patient (pat) and a healthy control proband (ctrl) were grown to subconfluence, and total protein was harvested with lysis buffer (150 mM NaCL, 1 mM EDTA, 1 % NP40, 20 mM Tris HCL, 0.25 % SDS, 1 mM Na_3_VO_4_, 10 mM NaF, 0.01 mM Na_2_MoO_4_). 75 μg total protein per lane was loaded on a 7,5 % TGX^TM^ Stain-Free FastCast^TM^ Acrylamide gel (Bio-Rad (Hercules, CA) propietary PAGE gel electrophoresis system) and electrophoresed at 160 V for 1.5 hrs. The Precision Plus Protein™ Standards for protein electrophoresis (Bio-Rad) were used as molecular weight ladder. The fluorescent blot image was taken using the ChemiDoc System (Bio-Rad). Gel-transfer onto a Trans-Blot® Turbo Mini LF PVDF membrane was carried out for 10 min at 25 V and 2.5 A using the Trans-Blot Turbo system (Bio-Rad). The blot was blocked with 5 % milk powder and incubated with a primary anti-MDA5 rabbit monoclonal antibody (Cell Signaling Technology; Cambridge, UK) at a 1:1000 dilution in Tris-buffered saline with Tween20 (TBST)/5 % milk powder at 4°C for 12 hrs and washed in TBST 3x5 min at 4°C. This was followed by incubation with a secondary goat anti-rabbit IgG horseradish peroxidase conjugated antibody at a 1:10,000 dilution in TBST/5 % milk powder and 3x5 min washes in TBST. Signals were detected using a standard enhanced chemiluminescence (ECL) protocol with an exposure time of 400 sec. Protein loading was checked by incubation with a primary anti-actin mouse antibody at a 1:10,000 dilution and a secondary anti-mouse goat antibody (SantaCruz). The ChemiDoc system (Bio-Rad) was used for quantification of IFIH1 relative to actin signal volume. IFIH1 signal volume for the control sample was set to 1, and the IFIH1 signal volume for the patient sample was corrected for equal loading as determined by comparing and adjusting actin signal volumes in control and patient samples.

# Supplementary Table S1

**Rare homozygous variants identified in this study.** Variants listed with bioinformatic assessments, (putative) functions of the respective proteins, data from animal models (if available) and associated monogenic disorders. MAF, minor allele frequency. HZ!, variants that have been documented as having occured homozygously in the ExAC database (http://exac.broadinstitute.org). MutTast, MutationTaster ([Schwarz et al., 2010](#_ENREF_10)): Poly, categorized as polymorphism. DC, categorized as disease-causing. CADD, CADD_phred (a score of greater or equal 20 indicates the 1% most deleterious and so on) (<http://cadd.gs.washington.edu/info)> ([Kircher et al., 2014](#_ENREF_6)). PPH, Polyphen2_HVAR_score (Polyphen2 score based on HumVar): prediction categories are probably damaging, possibly damaging, or benign, along with a numerical score ranging from 0.0 (benign) to 1.0 (damaging). SIFT([Flanagan et al., 2010](#_ENREF_3)): close to zero is most damaging. All of these scores have been retrieved from the dbNSFP database ([Liu et al., 2013](#_ENREF_8)) (<https://sites.google.com/site/jpopgen/dbNSFP> ). ar, autosomal recessive. ad, autosomal dominant.

**Supplementary Table S2**

**Blood count and immunological evaluation.** CBC, complete blood count; RBC, red blood cell count; Hb, hemoglobin; Hct, hematocrit; MCV, mean corpuscular volume; MCH, mean corpuscular hemoglobin; MCHC, Mean corpuscular hemoglobin concentration; Pl, platelets; WBCs, white blood cells;

**3 Supplementary Figure S1**

**Clinical presentation of patient II:1.** (**a**) Patient II:1 at the age of 2 years and (**b**,**c**) 5 ^8^/_12_ years. Note the severe microcephaly, broad nose, long philtrum and low set prominent ears. (**d**) Brain MRI (axial T1) and (**e**) (sagittal T1) at the age of 1 year. Note the simplified gyral pattern, deep sylvian fissures, prominent basal ganglia, hypoplastic corpus callosum, but normal cerebellum. (**f–h**) Cranial CT brain without contrast at the age of 5 years, showing prominent fourth ventricle, anterior interhemispheric fissure and abnormal gyral pattern with cortical atrophic changes.

**3 References for Supplementary Material**

Beck, B.B., Phillips, J.B., Bartram, M.P., Wegner, J., Thoenes, M., Pannes, A., Sampson, J., Heller, R., Gobel, H., Koerber, F., Neugebauer, A., Hedergott, A., Nurnberg, G., Nurnberg, P., Thiele, H., Altmuller, J., Toliat, M.R., Staubach, S., Boycott, K.M., Valente, E.M., Janecke, A.R., Eisenberger, T., Bergmann, C., Tebbe, L., Wang, Y., Wu, Y., Fry, A.M., Westerfield, M., Wolfrum, U., and Bolz, H.J. (2014). Mutation of POC1B in a severe syndromic retinal ciliopathy. *Hum Mutat* 35**,** 1153-1162.

Elsayed, S.M., Phillips, J.B., Heller, R., Thoenes, M., Elsobky, E., Nurnberg, G., Nurnberg, P., Seland, S., Ebermann, I., Altmuller, J., Thiele, H., Toliat, M., Korber, F., Hu, X.J., Wu, Y.D., Zaki, M.S., Abdel-Salam, G., Gleeson, J., Boltshauser, E., Westerfield, M., and Bolz, H.J. (2015). Non-manifesting AHI1 truncations indicate localized loss-of-function tolerance in a severe Mendelian disease gene. *Hum Mol Genet* 24**,** 2594-2603.

Flanagan, S.E., Patch, A.M., and Ellard, S. (2010). Using SIFT and PolyPhen to predict loss-of-function and gain-of-function mutations. *Genet Test Mol Biomarkers* 14**,** 533-537.

Fu, W., O'connor, T.D., Jun, G., Kang, H.M., Abecasis, G., Leal, S.M., Gabriel, S., Rieder, M.J., Altshuler, D., Shendure, J., Nickerson, D.A., Bamshad, M.J., Project, N.E.S., and Akey, J.M. (2013). Analysis of 6,515 exomes reveals the recent origin of most human protein-coding variants. *Nature* 493**,** 216-220.

Kawalia, A., Motameny, S., Wonczak, S., Thiele, H., Nieroda, L., Jabbari, K., Borowski, S., Sinha, V., Gunia, W., Lang, U., Achter, V., and Nurnberg, P. (2015). Leveraging the power of high performance computing for next generation sequencing data analysis: tricks and twists from a high throughput exome workflow. *PLoS One* 10**,** e0126321.

Kircher, M., Witten, D.M., Jain, P., O'roak, B.J., Cooper, G.M., and Shendure, J. (2014). A general framework for estimating the relative pathogenicity of human genetic variants. *Nat Genet* 46**,** 310-315.

Li, H., and Durbin, R. (2009). Fast and accurate short read alignment with Burrows-Wheeler transform. *Bioinformatics* 25**,** 1754-1760.

Liu, X., Jian, X., and Boerwinkle, E. (2013). dbNSFP v2.0: a database of human non-synonymous SNVs and their functional predictions and annotations. *Hum Mutat* 34**,** E2393-2402.

Mckenna, A., Hanna, M., Banks, E., Sivachenko, A., Cibulskis, K., Kernytsky, A., Garimella, K., Altshuler, D., Gabriel, S., Daly, M., and Depristo, M.A. (2010). The Genome Analysis Toolkit: a MapReduce framework for analyzing next-generation DNA sequencing data. *Genome Res* 20**,** 1297-1303.

Schwarz, J.M., Rodelsperger, C., Schuelke, M., and Seelow, D. (2010). MutationTaster evaluates disease-causing potential of sequence alterations. *Nat Methods* 7**,** 575-576.

Via, M., Gignoux, C., and Burchard, E.G. (2010). The 1000 Genomes Project: new opportunities for research and social challenges. *Genome Med* 2**,** 3.

Yeo, G., and Burge, C.B. (2004). Maximum entropy modeling of short sequence motifs with applications to RNA splicing signals. *J Comput Biol* 11**,** 377-394.
