# Supplementary material for: Recurrent and Prolonged Infections in a Child with a Homozygous IFIH1 Nonsense Mutation
Source: Front Genet. 2017 Sep 22;8:130. doi: 10.3389/fgene.2017.00130 (PMC5614965; doi:10.3389/fgene.2017.00130)
Supplement: Supplementary file 2 [file Data_Sheet_2.doc]

Zaki et al.: SUPPLEMENTARY MATERIAL

**Recurrent and prolonged infections in a child with a homozygous *IFIH1* nonsense mutation**

Maha Zaki^1^, Michaela Thoenes^2^, Amit Kawalia^3^, Peter Nürnberg^3,4^, Rolf Kaiser^5^, Raoul Heller^2^, Hanno J. Bolz^2^*

^1^Clinical Genetics Department, Human Genetics and Genome Research Division, National Research Centre, Cairo, Egypt.

^2^Institute of Human Genetics, University Hospital of Cologne, Cologne, Germany.

^3^Cologne Center for Genomics, University of Cologne, Cologne, Germany.

^4^Cologne Excellence Cluster on Cellular Stress Responses in Aging-Associated Diseases (CECAD), University of Cologne, Cologne, Germany.

^5^Institute of Virology, University Hospital of Cologne, Cologne, Germany.

^6^Bioscientia Center for Human Genetics, Ingelheim, Germany.

*Correspondence to: Hanno J. Bolz, Institute of Human Genetics, University Hospital of Cologne, Kerpener Str. 34, Cologne, Germany. [hanno.bolz@uk-koeln.de](mailto:hanno.bolz@uk-koeln.de)

| **Chr** | **HGNC** | **RefSeq** | **Function** | **cDNA** | **Protein** | **Var. name** | **MAF %**  (ExAC) | **Mut**  **Tast** | **PPH** | **SIFT** | **CADD** | **Animal models**  (homozygous k.o./LoF) | **Mendelian**  **Disease** |
| --- | --- | --- | --- | --- | --- | --- | --- | --- | --- | --- | --- | --- | --- |
| 1 | *CCNL2* | NM_001039577.3 | splicing, apoptosis | c.278G>C | p.R93P | rs200316100 | 0.58 | DC | 0.99 | 0.01 | 15.52 | --- | --- |
|  | *NOL9* | NM_024654.4 | Hematopoiesis,  pancreas  morphogenesis | c.239C>T | p.T80I | rs79736441 | 0.86  1xHZ! | Poly | 0.001 | 0.19 | 12.28 | hypoplastic pancreas, liver,  intestine; ↓hematopoietic cells,  erythrocytes, lymphocytes | --- |
|  | *RERE* | NM_012102.3 | apoptosis | c.3568_3573  delAAGGAG | p.Lys1190_  Glu1191del | --- | 0.4  3xHZ! | DC | --- | --- | --- | lethality, severe malformations | --- |
|  | *DFFA* | NM_213566.1 | apoptosis | c.493T>C | p.C165R | rs78046004 | 0.32  6xHZ! | Poly | 0.161 | 0.82 | 5.186 | --- | --- |
|  | *KIAA2013* | NM_138346.2 | ? | c.64C>T | p.L22F | rs552116013 | 0.19 | Poly | 0.858 | 0.11 | 18.76 | --- | --- |
|  | *AADACL3* | NM_001103170.1 | arylacetamide  deacetylation? | c.1001C>T | p.P334L | rs138344989 | 0.03  1xHZ! | DC | 0.799 | 0.14 | 14.19 | --- | --- |
|  | *DNAJC16* | NM_015291.2 | ? | c.442C>A | p.H148N | --- | --- | Poly | 0.322 | 0.38 | 17.72 | --- | --- |
|  | *SPEN* | NM_015001.2 | transcriptional  repressor | c.607C>G | p.P203A | --- | 0.001 | DC | 0.023 | 0.01 | 13.06 | embryonically lethal | --- |
|  | *HSPG2* | NM_005529.5 | extracellular matrix | c.9719C>T | p.A3240V | rs62642505 | 0.28  7xHZ! | Poly | 0.027 | 0.39 | 4.997 | embryonically lethal or severe  skeletal defects | Schwartz-Jampel  syndrome (a.r.) |
|  |  |  |  | c.3292G>A | p.A1098T | rs2501264 | 0.25  4xHZ! | Poly | 0.01 | 0.41 | 3.739 |  |  |
|  | *TESK2* | NM_007170.2 | spermiogenesis | c.479T>C | p.I160T | rs368533762 | 0.001 | DC | 0.934 | 0.02 | 29.1 | --- | --- |
|  | *BEND5* | NM_024603.2 | ? | c.733C>G | p.R245G | --- | --- | DC | 0.972 | --- | 16.52 | --- | --- |
|  | *PHGDH* | NM_006623.3 | 3-phosphoglycerate  dehydrogenase | c.1273G>A | p.V425M | rs121907988 | 0.001 | DC | 0.674 | 0.02 | 24 | 3-PHGDH deficiency | 3-PHGDH deficiency  (a.r.) |
|  | *LOR* | NM_000427.2 | cornified  cell envelope | c.272C>T | p.S91F | rs533773105 | 1.8 | Poly | 0.528 | 0 | 0.702 |  | Vohwinkel syndrome,  progressive symm. ery-  throkeratoderma (a.d.) |
|  | *ARHGEF11* | NM_198236.1 | Rho-dependent  signaling | c.4634C>G | p.S1545C | rs148989479 | 0.01 | Poly | 0.002 | 0.02 | 12.75 | no obvious phenotype | --- |
|  | *C1orf204* | NM_001134233.1 | ? | c.466G>A | p.A156T | rs61742098 | 0.06 | Poly | 0.002 | 0 | 7.271 | --- | --- |
|  | *IGSF9* | NM_001135050.1 | immunoglobulin  superfamily;  cell adhesion? | c.3078T>A | p.S1026R | --- | --- | DC | 0.725 | --- | 16.34 | k.o. mice are are viable  and fertile | --- |
|  | *CD244* | NM_016382.3 | cell surface receptor  on natural killer cells | c.654G>T | p.Q218H | rs184282870 | 0.004 | Poly | 0.006 | 0.15 | 10.5 | ↓NK cell cytolysis in k.o. mice | --- |
|  | *TOMM40L* | NM_032174.4 | mitochondrial import  receptor subunit | c.530T>G | p.V177G | rs202137061 | 0.89 | DC | 0.021 | 0.03 | 17.77 | --- | --- |
|  | *KIF21B* | NM_001252103.1 | microtubule-based  motor | c.3382A>C | p.T1128P | rs200705792 | 1.9 | Poly | 0.029 | 0.14 | 13.74 | k.o. mice: ↓learning+memory | --- |
| 2 | *PLA2R1* | NM_007366.4 | phospholipase A2  receptor | c.2680G>A | p.V894M | --- | --- | Poly | 0.497 | 0.16 | 16.49 | k.o. mice: viable, fertile | --- |
|  | *IFIH1* | NM_022168.2 | cytoplasmic sensor  for viral double-  stranded RNAs | c.2665A>T | p.K889* | --- | --- | DC | --- | 1 | 42 | k.o. mice have impaired  immune defense | Aicardi-Goutieres- and  Singleton-Merten  syndrome (both a.d.) |
| 3 | *SEMA5B* | NM_001256347.1 | axon growth | c.2519T>C | p.V840A | rs2276782 | --- | Poly | --- | 0.12 | 2.948 | k.o. mice have defects in  arborization of retinal cells | --- |
|  | *IGSF10* | NM_178822.4 | immunoglobulin  superfamily;  cell adhesion? | c.345T>G | p.N115K | --- | --- | DC | 1 | 0 | 17.96 | --- | delayed puberty (a.d.)? |
|  | *PIGZ* | NM_025163.2 | anchors proteins to  blood cells’ surface | c.371C>T | p.A124V | rs144088027 | 0.52  1xHZ! | Poly | 0.002 | 0.77 | 1.154 | --- | --- |
| 4 | *ACSL1* | NM_001995.2 | long-chain fatty acids  metabolism | c.1958T>G | p.V653G | rs200382149 | 1.11 | DC | 0.91 | 0 | 19.38 | k.o. mice: ↓liver acyl-CoA | --- |
| 6 | *FOXQ1* | NM_033260.3 | cell differentiation,  tumorigenesis | c.1160T>G | p.V387G | rs561503035 | 1.5  1xHZ! | Poly | 0 | 0.44 | 0.518 | k.o. mice have increased  embryonic lethality | --- |
| 7 | *AGAP3* | NM_031946.4 | synaptic plasticity,  AMPA receptor  trafficking | c.94T>G | p.C32G | --- | --- | Poly | 0 | 0.71 | 9.845 | --- | --- |
| 9 | *FRRS1L* | NM_014334.2 | controls gating of  AMPAR complex | c.151C>G | p.R51G | rs10979724 | --- | Poly | --- | 0.11 | --- | --- | --- |
| 10 | *PDCD11* | NM_014976.1 | rRNA maturation,  18S rRNA generation | c.2095G>A | p.E699K | [rs61751510](http://www.ncbi.nlm.nih.gov/projects/SNP/snp_ref.cgi?rs=rs61751510) | 0.51 | Poly | 0.004 | 0.99 | 3.464 | --- | --- |
| 15 | *CSPG4* | NM_001897.4 | cell-substratum  interactions in early  melanoma spreading | c.5108G>C | p.R1703P | --- | --- | DC | 0.222 | 0.29 | 18.71 | k.o. mice: abnormal dentate  gyrus morphology + smooth  muscle cell physiology | --- |
| 16 | *CACNA1H* | NM_021098.2 | voltage-dependent  Ca^2+^ channel | c.1702G>A | p.D568N | rs61056448 | 0.54  1xHZ! | Poly | 0.007 | 0.24 | 3.248 | --- | hypertension +primary  aldosteronism (a.d.) |
|  | *CPPED1* | NM_018340.2 | glucose uptake in  adipocytes | c.418G>A | p.E140K | [rs149102231](http://www.ncbi.nlm.nih.gov/projects/SNP/snp_ref.cgi?rs=rs149102231) | 0.64  13xHZ! | Poly | 0.009 | 0.92 | 0.678 | --- | --- |
|  | *ABCC1* | NM_004996.3 | ATP-binding cassette (ABC) transporter | c.250G>C | p.V84L | --- | --- | Poly | 0.005 | 0.57 | 12.49 | homozygous mice:↓inflamma-  tory response, ↓metabolism | --- |
|  | *USP31* | NM_020718.3 | ubiquitin specific  peptidase 31 | c.1513A>T | p.T505S | [rs61760226](http://www.ncbi.nlm.nih.gov/projects/SNP/snp_ref.cgi?rs=rs61760226) | 0.12 | DC | 0.045 | 0.62 | 11.17 | --- | --- |
|  | *KIFC3* | NM_005550.3 | microtubule-based  motor | c.856G>A | p.V286M | rs534173077 | --- | DC | 0.901 | 0.21 | 24.5 | k.o. mice: normal | --- |
|  | *CCDC113* | NM_014157.3 | ciliogenesis; centro-  some component | c.539G>A | p.R180H | --- | 0.0008 | DC | 0.037 | 0.16 | 11.34 | --- | --- |
|  | *SLC12A4* | NM_001145962.1 | K^+^/Cl^-^ cotransport;  erythroid maturation? | c.52A>G | p.T18A | --- | --- | Poly | --- | 0.57 | 3.425 | --- | --- |
|  | *DPEP3* | NM_022357.3 | metabolism of various  dipeptides | c.988G>A | p.D330N | [rs113700215](http://www.ncbi.nlm.nih.gov/projects/SNP/snp_ref.cgi?rs=rs113700215) | 0.71 | DC | 0.991 | 0.01 | 24 | --- | --- |
|  | *ADAT1* | NM_012091.3 | tRNA adenosine  deaminase 1 | c.46G>A | p.G16R | rs202191887 | 0.04 | Poly | 0.002 | 0.84 | 1.705 | --- | --- |
| 17 | *SLC46A1* | NM_080669.4 | folate transporter | c.1241A>C | p.Y414S | --- | 0.48 | DC | 0.998 | 0 | 28 | k.o. mice: ↑homocysteine | hereditary folate  malabsorption (a.r.) |
|  | *TBX4* | NM_018488.2 | hindlimb development | c.941C>G | p.A314G | --- | --- | Poly | 0.07 | --- | 12.26 | k.o. mice: lack hindlimb buds,  die in embryonic phase | small patella syndrome  (a.d.) |
|  | *RGS9* | NM_003835.3 | phototransduction | c.773C>T | p.S258L | [rs12452285](http://www.ncbi.nlm.nih.gov/projects/SNP/snp_ref.cgi?rs=rs12452285) | 0.95  28xHZ! | Poly | 0.004 | 0.39 | 8.002 | k.o. mice: delayed recovery of  photoresponse | bradyopsia (a.r.) |
|  | *HELZ* | NM_014877.3 | RNA helicase | c.5407T>C | p.S1803P | --- | --- | Poly | 0.121 | 0.04 | 11.91 | k.o. mice: normal | --- |
|  | *ABCA9* | NM_080283.3 | membrane transport | c.863T>C | p.I288T | [rs61744902](http://www.ncbi.nlm.nih.gov/projects/SNP/snp_ref.cgi?rs=rs61744902) | 0.24  4xHZ! | Poly | 0.248 | 0.03 | 11.08 | --- | --- |
|  | *TMC6* | NM_007267.6 | transmembrane  channel? | c.847G>A | p.G283S | rs201267987 | 0.07 | Poly | 0.047 | 1 | 9.695 | --- | epidermodysplasia  verruciformis (a.r.) |
| 19 | *ACTN4* | NM_004924.4 | actin-binding | c.1963G>C | p.A655P | --- | 0.007 | DC | 0.991 | 0.05 | 16.38 | k.o. mice: proteinuria,  glomerular disease,  early death | focal+segmental  glomerulosclerosis  (FSGS; a.d.) |
| 22 | *GALR3* | NM_003614.1 | galanin receptor 3 | c.490G>C | p.V164L | rs201220537 | 0.32  1xHZ! | DC | 0.013 | 0.35 | 11.61 | male k.o. mice: ↑cholesterol +  triglyceride | --- |
| X | *KIF4A* | NM_012310.4 | microtubule-based  motor | c.3547C>T | p.P1183S | --- | 0.01  7xHZ! | Poly | 0.004 | --- | 1.251 | hemizygous mice: normal | mental retardation  (X-linked) |

**SUPPLEMENTARY TABLE S1**

**Rare homozygous variants identified in this study.** Variants listed with bioinformatic assessments, (putative) functions of the respective proteins, data from animal models (if available) and associated monogenic disorders. MAF, minor allele frequency. HZ!, variants that have been documented as having occured homozygously in the ExAC database (http://exac.broadinstitute.org). MutTast, MutationTaster[^1^](#_ENREF_1): Poly, categorized as polymorphism. DC, categorized as disease-causing. CADD, CADD_phred (a score of greater or equal 20 indicates the 1% most deleterious and so on) (<http://cadd.gs.washington.edu/info>)[^2^](#_ENREF_2). PPH, Polyphen2_HVAR_score (Polyphen2 score based on HumVar): prediction categories are probably damaging, possibly damaging, or benign, along with a numerical score ranging from 0.0 (benign) to 1.0 (damaging). SIFT[^3^](#_ENREF_3): close to zero is most damaging. All of these scores have been retrieved from the dbNSFP database[^4^](#_ENREF_4) (<https://sites.google.com/site/jpopgen/dbNSFP> ). ar, autosomal recessive. ad, autosomal dominant.

|  | **Patient** | **Normal Range** |
| --- | --- | --- |
| **CBC**  RBC  Hb  Hct  MCV  MCH  MCHC  Pl  WBCs  Basophils  Eosinophils  Staff cells  Segmented  Lymphocytes  Monocytes | 4.6  11.4  34  75  25  34  121  9.9  0  5  3  32  56  4 | 4.0 - 5.2 10^6^/cmm  11 -14 gm/dl  36 - 47 %  75 - 87 fl  24 - 30 pg  31 - 37 gm/dl  150 - 450 10^3^/cmm  5 - 15 10^3^/cmm  0 - 2 %  1 - 6 %  0 - 6 %  30 - 50 %  35 - 60 %  1 - 10 % |
| **Immunoglobulins**  IgG  IgG1  IgG2  IgG3  IgG4  IgM  IgA | 11.3  8.6  1.1  >1.22  <0.004  2  1.7 | g/l  6.460 - 14.510  2.880 - 9.180  0.440 - 3.750  0.155 - 0.853  0.004 - 0.992  0.550 - 2.320  0.280 - 2.220 |
| **Lymphocyte subsets**  CD3  CD19  CD16  CD4  CD8 | 65.3  14.9  13.5  34.1  17.4 | %  60 - 67  13 - 27  4 - 17  31 - 47  18 - 35 |

**SUPPLEMENTARY TABLE S2**

**Blood count and immunological evaluation.** CBC, complete blood count; RBC, red blood cell count; Hb, hemoglobin; Hct, hematocrit; MCV, mean corpuscular volume; MCH, mean corpuscular hemoglobin; MCHC, Mean corpuscular hemoglobin concentration; Pl, platelets; WBCs, white blood cells;

**References**

1 Schwarz, J. M., Rodelsperger, C., Schuelke, M. & Seelow, D. MutationTaster evaluates disease-causing potential of sequence alterations. *Nat Methods* 7, 575-576, doi:10.1038/nmeth0810-575 (2010).

2 Kircher, M. *et al.* A general framework for estimating the relative pathogenicity of human genetic variants. *Nat Genet* 46, 310-315, doi:10.1038/ng.2892 (2014).

3 Flanagan, S. E., Patch, A. M. & Ellard, S. Using SIFT and PolyPhen to predict loss-of-function and gain-of-function mutations. *Genet Test Mol Biomarkers* 14, 533-537, doi:10.1089/gtmb.2010.0036 (2010).

4 Liu, X., Jian, X. & Boerwinkle, E. dbNSFP v2.0: a database of human non-synonymous SNVs and their functional predictions and annotations. *Hum Mutat* 34, E2393-2402, doi:10.1002/humu.22376 (2013).
