# Supplementary figures and images for: Recurrent and Prolonged Infections in a Child with a Homozygous IFIH1 Nonsense Mutation
Source: Front Genet. 2017 Sep 22;8:130. doi: 10.3389/fgene.2017.00130 (PMC5614965; doi:10.3389/fgene.2017.00130)

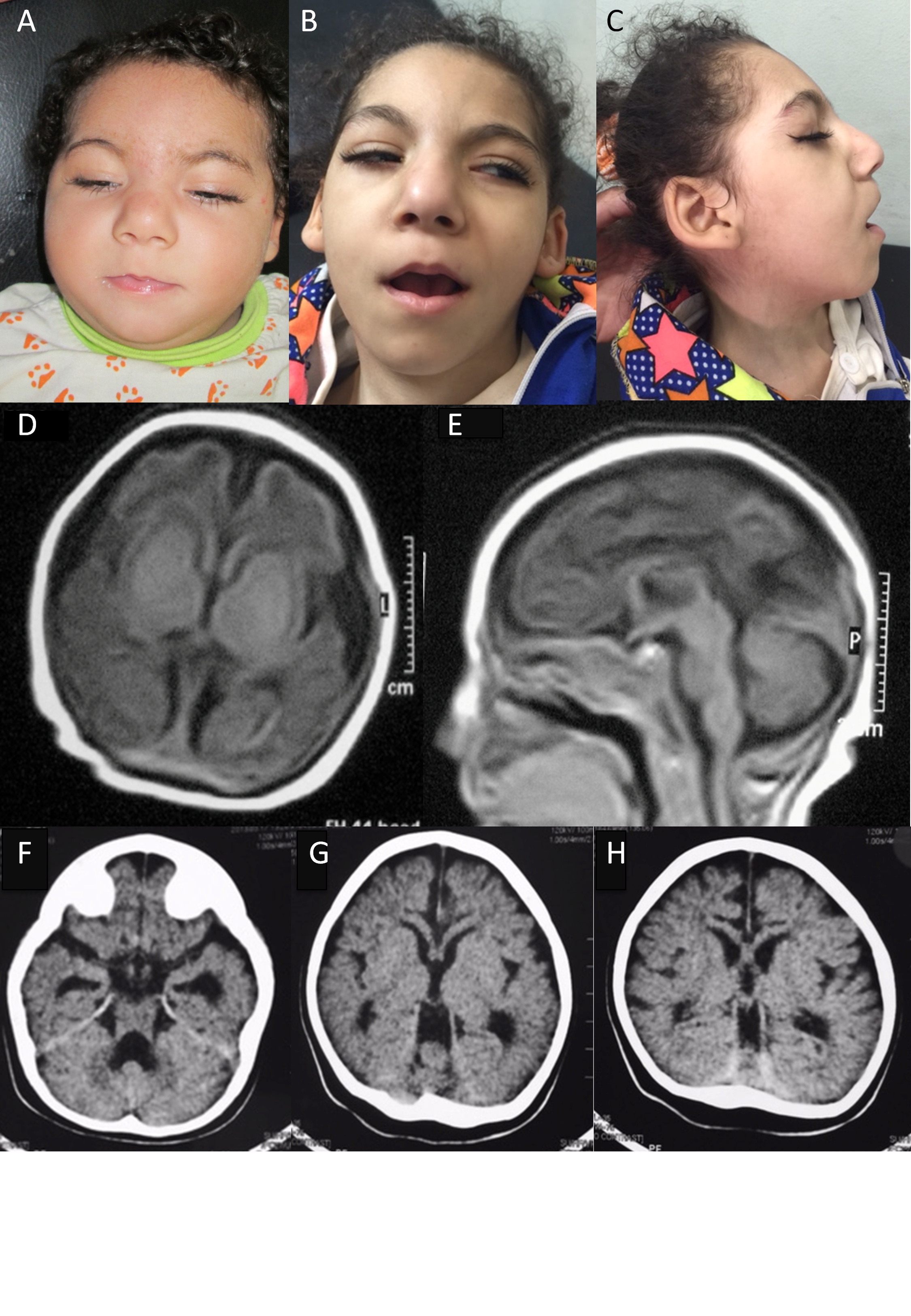

Supplement: Supplementary file 3 [file Image_1.JPEG]
